# Respectful maternity care interventions to address women mistreatment in childbirth: What has been done?

**DOI:** 10.1186/s12884-024-06524-w

**Published:** 2024-04-26

**Authors:** Pablo Mira-Catalá, Ildefonso Hernández-Aguado, Elisa Chilet-Rosell

**Affiliations:** 1 Public Health Department, Miguel Hernández University, 03550 Alicante, Spain; 2 CIBER de Epidemiología y Salud Pública (CIBERESP), 28029 Madrid, Spain

**Keywords:** Respectful maternity care, Mistreatment, Disrespect and abuse, Obstetrical violence, Obstetrics, Childbirth, Reproductive rights, Human rights, Interventions

## Abstract

**Introduction:**

Over the last decade, there has been an increasing number of studies regarding experiences of mistreatment, disrespect and abuse (D&A) during facility-based childbirth. These negative experiences during labour have been proven to create a barrier for seeking both facility-based childbirth and postnatal health care, as well as increasing severe postpartum depression among the women who experienced them. This constitutes a serious violation of human rights. However, few studies have carried out specifically designed interventions to reduce these practices. The aim of this scoping review is to synthetise available evidence on this subject, and to identify initiatives that have succeeded in reducing the mistreatment, D&A that women suffer during childbirth in health facilities.

**Methods:**

A PubMed search of the published literature was conducted, and all original studies evaluating the efficacy of any type of intervention specifically designed to reduce these negative experiences and promote RMC were selected.

**Results:**

Ten articles were included in this review. Eight studies were conducted in Africa, one in Mexico, and the other in the U.S. Five carried out a before-and-after study, three used mixed-methods, one was a comparative study between birth centres, and another was a quasi-experimental study. The most common feature was the inclusion of some sort of RMC training for providers at the intervention centre, which led to the conclusion that this training resulted in an improvement in the care received by the women in childbirth. Other strategies explored by a small number of articles were open maternity days, clinical checklists, wall posters and constant user feedback.

**Discussion:**

These results indicate that there are promising interventions to reduce D&A and promote RMC for women during childbirth in health facilities. RMC training for providers stands as the most proven strategy, and the results suggest that it improves the experiences of care received by women in labour.

**Conclusion:**

The specific types of training and the different initiatives that complement them should be evaluated through further scientific research, and health institutions should implement RMC interventions that apply these strategies to ensure human rights-based maternity care for women giving birth in health facilities around the world.

## Abstract (plain language summary)

Women giving birth experience mistreatment, disrespect, and abuse during labour in health facilities around the world, which constitutes a serious human rights violation. This scoping review synthetises all available information on interventions specifically designed to reduce these negative experiences and to promote respectful maternity care. Although research in this regard is still scarce and focused in low-resource countries, our results indicate that there are promising initiatives to tackle this phenomenon. The most tested strategy is respectful maternity care training for providers, the results of which suggest that it is successful in improving the experiences of care received by women in labour. Other different strategies have been explored, such as open maternity days, clinical checklists, wall posters and constant user feedback. This review shows that there are promising interventions to reduce disrespect and abuse and promote respectful maternity care for women during facility-based childbirth, which should be implemented by health institutions to ensure human-rights based maternity care for women giving birth in health facilities around the world.

## Introduction

Over the last decade, there has been an increasing number of studies worldwide regarding experiences of mistreatment, disrespect and abuse (D&A) during facility-based childbirth [1]. These negative experiences during labour have been proven to create a barrier for seeking both facility-based childbirth and postnatal health care, as well as increasing fear of childbirth and severe postpartum depression among the women who experienced them [2, 3].

This is not only a quality-of-care issue, but also constitutes a serious violation of human rights. Every woman has the right to the highest attainable level of health, including the right to respectful health care during pregnancy and labour, as stated by the Assertion of Universal Rights of Childbearing Women [4].

It is important to note that these behaviours by healthcare providers are by definition not intentional and may overlap with other respectful care practices. Nevertheless, women’s experiences of D&A should be considered as such regardless of intentionality. In addition, the characteristics of the healthcare system may explain some of these negative experiences, but should not be used as justification for this mistreatment of women [5].

Many of the evaluations of D&A during childbirth were initially carried out in low-resource settings. Systematic reviews and meta-analysis in Africa and India have estimated its prevalence at 44% and 71%, respectively [6, 7]. However, childbearing women from middle and high-resource countries have also reported mistreatment and D&A during labour. In Latin America, two national surveys in Mexico and Ecuador have described prevalence rates higher than 30% [8, 9]. Similar research in the U.S. has reported results over 17% [10], ranging up to 27%-54% in the Netherlands [11, 12], and 38%-67% in Spain [13, 14]. However, it is not possible to compare these prevalence studies, as different definitions are used to assess D&A in each of them.

The need for standardised typology and operational definitions of this phenomenon impedes wider research in this area [5]. In 2010, Bowser and Hill reported seven types of disrespectful and abusive practices during childbirth: physical abuse, non-consented care, non-confidential care, non-dignified care, discrimination, abandonment, and detention in health facilities [15]. In 2015, Bohren et al. suggested the term “mistreatment of women”, since they believed it to be broader and more inclusive for the complete range of negative experiences described in the literature. In their systematic review, they also proposed a new categorisation system: physical abuse, sexual abuse, verbal abuse, stigma and discrimination, failure to meet professional standards of care, poor rapport between women and providers, and health system conditions and constraints [5].

In Latin America, discussions have not focused on D&A, but rather on terminology referring to “obstetric violence” as one of the various types of violence against women [16].

Gender inequalities have been fundamental to the conceptualisation of this term. In this regard, Nagle et al. observed a significant relationship between structural sexism and C-section rates in the U.S. [17]. This finding is in line with the theoretical framing that categorizes it as being a symptom of structural violence and sexism towards women.

Sadler et al. proposed that obstetric violence as a term could address these structural determinants of violence. One reason why this term is not more widely used is that healthcare providers are resistant to the use of the concept of violence [18]. Focusing the debate on individual malpractices can give rise to unproductive hostility, which is why it is a priority to avoid blaming health professionals as a group [19]. With this in mind, we will refer to these negative experiences of childbirth using the terms noted above (mistreatment and D&A) and avoid using the term obstetric violence.

Based on the principle that the absence of D&A alone is not enough, respectful maternity care (RMC) is an alternative approach which also highlights the rights of women, promotes equitable access to evidence-based practices and recognises the unique needs and preferences of women. This initiative has been recommended by the WHO as an approach to care for a positive childbirth experience [20].

Shakibazadeh et al. described some of the concepts that constitute RMC [21]. Jolivet et al. operationalised these concepts into seven human rights-based categories of RMC: the right to be free from harm and ill treatment; the right to dignity and respect; the right to information, informed consent and respect for choices and preferences (including the right to companionship of choice wherever possible); the right to privacy and confidentiality; the right to non-discrimination, equality and equitable care; the right to timely healthcare and to the highest attainable level of health; and the right to liberty, autonomy, self-determination and freedom from coercion [22]. Both respectful and disrespectful care should be taken into account, given that some practices may not seem very disrespectful but should not be considered acceptable as part of respectful maternity care [23].

Women’s healthcare should be based on the best available scientific evidence, subject to systematic review and adapted to each patient’s preferences, respecting their rights and principles. This evidence-based approach supports safe, effective and individualised care, while avoiding inappropriate or unnecessarily risky interventions that do not benefit women´s health [24].

Identifying successful interventions that have addressed these negative experiences during childbirth or that have been directed towards improving RMC may help to design and implement interventions based on best practice in other maternity services and countries. The aim of this article is to summarise the available evidence regarding the initiatives that have been taken to eradicate the mistreatment and D&A that women undergo during childbirth and to promote RMC in health facilities worldwide.

## Methods

### Study design

We conducted a descriptive scoping review of the available peer-reviewed literature. We followed the Arksey and O’Malley’s five-stage framework [25]. Research was conducted to answer the following question: What interventions have been proven as effective to reduce mistreatment, D&A during facility-based childbirth?

### Search strategy

To identify relevant articles, published literature was searched in PubMed using Mesh and free-text terms referring to two main concepts: mistreatment of women and obstetrics.

The search formula was: *“Obstetric violence” OR ((“Violence”[Mesh] OR “Gender-Based Violence”[Mesh] OR “Dehumanization”[Mesh] OR “Human Rights”[Mesh] OR “Human Rights Abuses”[Mesh] OR “Physical Abuse”[Mesh] OR “Emotional Abuse”[Mesh] OR “Malpractice”[Mesh] OR “Health Services Misuse”[Mesh] OR “Disrespect” OR “Disrespectful” OR “Respectful” OR “Mistreatment” OR “Abuse” OR “Medicalization” OR “Industrialization”) AND (“Delivery, Obstetric”[Mesh] OR “Parturition”[Mesh] OR “Obstetrics”[Mesh])).*

The “Abstract” search filter was used (see “Eligibility Criteria”).

No year restrictions were applied. Any article published previously to the date of the search was included in the review. The search was conducted on June 7, 2022.

### Eligibility criteria

We selected any original study that assessed the effectiveness of interventions specifically designed to reduce experiences of mistreatment and D&A or to promote RMC during facility-based childbirth. Both clinical and institutional interventions were included.

The concepts mistreatment and D&A were considered inherently as presented in the original studies that proposed these two terms, as detailed in the introduction.

Articles were selected in English, Spanish, French, Portuguese and Italian.

Articles without an abstract were excluded. We also discarded studies whose methodology was not explicitly detailed (study protocols, commentaries, and conferences).

According to the definition stated before, these negative experiences of care would also encompass medicalization of childbirth. This includes unnecessary C-sections and similar procedures. Nevertheless, the problem on these avoidable medical interventions was recognised decades before research started to focus on mistreatment and D&A as a continuum. Consequently, a large body of literature has been published to this respect, which will require specific reviews on this subject. Moreover, most of the studies regarding this question lack the mistreatment lens when analyzing this issue. For these reasons, articles that only evaluated initiatives to reduce unnecessary C-sections and comparable medical interventions were also excluded.

A particular case are the studies that exclusively analysed programs on the presence of a companion of choice during labor. We also discarded these articles in order not to interfere with the overall scope of the review, since these only evaluated the change on some concrete first-order theme.

### Study selection

The three authors participated in the study selection. Each abstract was screened by two different researchers. The same procedure was followed for the full-text evaluation, so that every article was selected by two researchers independently. Discrepancies during these two stages were discussed with the third author until consensus was reached.

### Data extraction

The following data were extracted: study type; target and objectives of the intervention (reducing mistreatment and D&A, increasing RMC); approach (quality of care, human rights, gender violence); description and scope of the intervention; evaluation methods; outcomes; and limitations and conclusion of the articles.

The selection of articles and data extraction were performed independently by two authors. Any discrepancies were resolved by consensus.

## Results

The initial search yielded 2,279 citations. After screening for their titles and abstract, 40 studies remained. Concordance reached 90%.

**Fig. 1:**
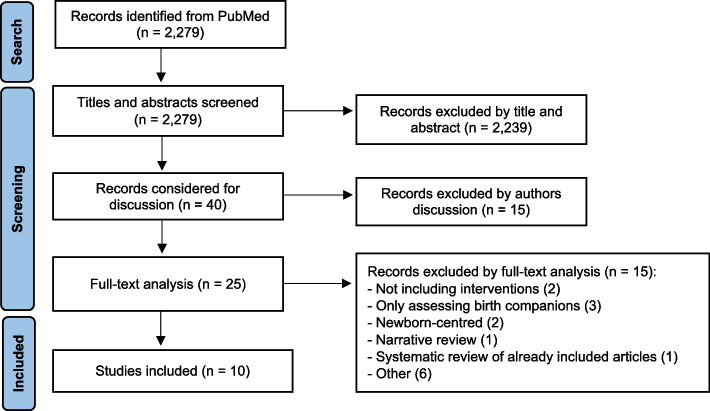
PRISMA flowchart of search and study inclusion process

After discussion, 15 additional articles were excluded. In case of any doubt, the article was considered for full-text analysis, prioritising the sensitivity of the search. Of the 25 articles that went through full-text analysis, 10 studies were finally included. No article was excluded for language reasons. This whole process is represented on Fig. 1.

The publication years ranged from 2015 to 2022, and all were located in Africa except for two, whose settings were Mexico [26] and the United States [27].

Of these 10 articles that were included, 5 did a before-and-after study [28–32], 3 used mixed-methods [26, 33, 34], one was a comparative study between birth centers [27], and another a quasi-experimental study [35]. Three of them focused on reducing D&A, and 5 on increasing RMC. One sought birth racial equity [27], and another aimed at humanised childbirth [33]. Every study approached this phenomenon as a quality-of-care issue, but only 5 of them addressed this topic from a human rights perspective (apart from the one approaching it as an ethnic disparity). Table 1 summarises the main characteristics of these articles.

**Table 1:** Summary characteristics of the studies that described RMC interventions

Reference	Study design	Objectives	Approach
Authors: Abuya et al. [28] Year: 2015 Setting: Kenya	Before-and-after	Reduce D&A	Human rights and quality-of-care issue
Authors: Afulani et al. [29] Year: 2019 Setting: Ghana	Before-and-after	Increase RMC	Quality-of-care issue
Authors: Almanza et al. [27] Year: 2022 Setting: United States	Comparative study between birth centers	Birth equity	Reducing ethnic disparities
Authors: Asefa et al. [30] Year: 2020 Setting: Ethiopia	Before-and-after	Increase RMC	Quality-of-care issue
Authors: Gélinas et al. [33] Year: 2022 Setting: Senegal	Mixed methods	Humanised childbirth	Human rights and quality-of-care issue
Authors: Kujawski et al. [31] Year: 2017 Setting: Tanzania	Before-and-after	Reduce D&A	Human rights and quality-of-care issue
Authors: Molina et al. [26] Year: 2019 Setting: Mexico	Mixed methods	Increase RMC	Quality-of-care issue
Authors: Oosthuizen et al. [32] Year: 2020 Setting: South Africa	Before-and-after	Increase RMC	Human rights and quality-of-care issue
Authors: Ratcliffe et al. [34] Year: 2016 Setting: Tanzania	Mixed methods	Reduce D&A	Human rights and quality-of-care issue
Authors: Smith et al. [35] Year: 2022 Setting: Zambia	Quasi-experimental	Increase RMC	Quality-of-care issue

*RMC* respectful maternity care, *D&A* disrespect and abuse

Most of the interventions were conducted at facility level with different action plans, none of the articles was designed as a policy or as a community-level approach.

The most common feature was to include some sort of RMC training for providers at the intervention center [26, 28–30, 32–35]. Four of them considered the implementation of D&A continuous feedback [28, 31, 32, 35], and another 3 were aimed at improving the infrastructure and/or available equipment [26, 31, 33]. Two of them proposed Maternity Open Days [28, 34], and another two, counselling for providers [28, 31]. One of them also included wall posters [30], another one, RMC checklists [26], and other, a provider-patient document on agreed behaviours during labour and delivery [35].

The article by Almanza et al. did not assess a concrete intervention but a comparison between Roots (a Black-owned culturally centred birth clinic) and other centers [27]. More detailed information about the studied interventions and the way they were evaluated is presented at Table 2.

**Table 2:** Interventions and results of the studies that described RMC interventions

Reference	Interventions	Evaluation	Results
Abuya et al. [28] 2015 Kenya	Policy level: continuous dialogue in technical meetings with government, civil society, and professional knowledge network Community level: training on RMC, community dialogue and counseling and resolution of reported cases by a mediator Facility level: training in RMC (including values clarification and attitude transformation), counselling for providers (supporting them with coping mechanisms to overcome experiences related to high workload, trauma or critic incidents), mentorship, Quality Improvement teams, Maternity Open Days and D&A monitoring	Interviews pre- and post-intervention (*n* = 641 and 728) and observations of provider-patient interactions during labour and delivery (*n* = 677 and 523, respectively)	Interviews: feelings of humiliation or disrespect decreased from 20 to 13% (*p* = 0.0004), physical abuse was reduced from 4 to 2% (*p* = 0.024); verbal abuse, from 18 to 11% (*p* = 0.01); violations of confidentiality, from 4 to 2% (*p* = 0.021); violations of privacy, from 7 to 6% (*p* = 0.101); and detainment, from 8 to 1% (*p* < 0.0001) Observations: physical abuse decreased from 3.8% to 0.4% (*p* = 0.003); violations of privacy during examination, from 34 to 13% *p* < 0.0001); and violations of privacy during delivery, from 92 to 79% *p* < 0.0001)
Afulani et al. [29] 2019 Ghana	Provider trainings based on methodology developed by PRONTO International: low‐tech, highly realistic simulation and team training with facilitated debriefing, to improve identification and management of obstetric and neonatal emergencies and team functioning	Interviews pre- and post-intervention (*n* = 215 and 318)	At baseline, 12% felt they were treated respectfully and 8% reported to be treated in a friendly manner, compared to 64% and 65% at endline (*p* < 0.01 for both differences) A relative increase of the full‐scale score on person-centered maternity care of 43%, with relative increases of 15% in dignity and respect, 87% in communication and autonomy and 45% in supportive care (all statistically significant, *p* < 0.001)
Almanza et al. [27] 2022 United States	Roots, where care is delivered by acknowledging the client’s cultural community as a strength, providing racially concordant care as able. It includes 13–15 prenatal visits (for no less than 30 min each) and 4 group classes. Postpartum care includes lactation support with 3 home visits in the first week, and clinic visits at week 2, 4, and 6	Comparing Roots (*n* = 80) to other BIPOC centers, using the sample of the GVtM (*n* = 2700)	Autonomy and respect scores were statistically higher for clients receiving culturally centered care at Roots. No statistical significance was found in scores between BIPOC and white clients, however there was a tighter range among BIPOC individuals, showing less variance in their experience of care
Asefa et al. [30] 2020 Ethiopia	Interactive training of service providers deploying various teaching methods (presentations, role plays, demonstrations, case studies, individual readings, video shows and a hospital visit). Placement of wall posters in labour rooms listing universal rights, describing manifestations of mistreatment and presenting guidelines on RMC	Post-partum interviews (n pre- and post-intervention = 198 and 190, respectively)	99.5% and 99% of women reported suffering at least one negative experience The number of mistreatment components experienced by women was reduced by 18% when the post-intervention group was compared with the pre-intervention group, adjusting for several clinical and sociodemographic variables (*p* < 0.05). Components: physical abuse, from 16.7% to 8.9% (*p* = 0.02); non-consented care, from 83.3% to 65.3% (*p* = < 0.001); refusal of preference, from 67.7% to 54.7% (*p* = 0.01). No significant difference was detected for verbal abuse, lack of information, privacy and confidentiality, and neglect and discrimination
Gélinas et al. [33] 2022 Senegal	Communication with communities, sharing the concept in health facilities, improving the working environment, evidence-based care practices and support development activities. Redesign of health facilities to provide natural birthing rooms with accessories (tatami mats, balls, cushions, swings, stepladders, and screens) and essential technical equipment. Staff training: 5S/Kaizen approach and evidence-based medicine with WHO’s standards for normal childbirth	Interviews (*n* = 20) and direct labour observations (average duration = 5 days/facility, *n* = 20)	Women who gave birth post-intervention appreciated their experience due to changes such as the opportunity to eat and drink, to be accompanied by a trusted person and to choose their position during childbirth. It was the way in which women were received at the health facility and the attitude of health professionals that were decisive in their level of satisfaction with care
Kujawski et al. [31] 2017 Tanzania	Maternity ward improvements, including moving the admissions area to a private room, using curtains for delivery and for physical examinations, posting supply stock outs to ensure transparency and build trust, and continuous customer satisfaction exit surveys. At facility management level, counseling of staff who continued to exhibit disrespectful behaviours and best practice sharing with other wards and the regional hospital	Interviews (*n* = 2983) before and after in two different facilities (the intervention and control group)	The intervention was associated with a 66% reduced odds of a woman experiencing D&A (*p* < 0.0001). The biggest reductions were for physical abuse (adjusted OR: 0.22, 0.05–0.97, *p* = 0.003) and neglect (0.36, 0.19–0.71, *p* = 0.045)
Molina et al. [26] 2019 Mexico	Implementation of an adapted version of the WHO Safe Childbirth Checklist with a mobile application to incorporate RMC (allowing birth companions, asking women about their preferred delivery position, and emphasizing clear communication regarding the care plan). Monthly clinical training courses for clinicians. Budget to fill supply gaps for essential medications and equipment, funds for gasoline to facilitate travel for women in need of referral and lodging in an existing patient hostel with food vouchers for pregnant women and their birth companion	Synchronised data of the mobile application (*n* = 384), and surveys (*n* = 221) and semi-structured interviews with birthing women (*n* = 28) and companions (*n* = 13)	384 (85.9%) women were attended by staff that used the adapted SCC during delivery. Adherence with offering a birth companion (OR: 3.06, 1.40–6.68, *p* < 0.01), free choice of birth position (2.75, 1.21–6.26, *p* = 0.02), and immediate skin-to-skin contact (4.53, 1.97–10.39, *p* < 0.01) improved 6–8 months after implementation. The 221 respondents of the survey were highly satisfied with their experience at the hospital, with a median satisfaction score of 10/10 versus 9/10 for the previous delivery. The prevalent narrative was that quality of care at the hospital had improved over time, and women were satisfied
Oosthuizen et al. [32] 2020 South Africa	CLEVER package: Clinical care and obstetric triage, Labour ward management to resolve the withholding of care, Elimination of barriers to meet basic human needs, Verification of care (monitoring, evaluation and feedback), Emergency obstetric simulation training, and RMC to improve birthing women’s experiences; implemented with a period for creating awareness and a core group of activities aimed at behavioural change	Interviews before (*n* = 653) and after (*n* = 679) in 10 different facilities (5 intervention sites and 5 control comparisons)	For consent to examination, being spoken nicely and treated respectfully during labour, and being satisfied with the treatment received, there were significant positive changes from baseline to end-line regarding the intervention group units (OR: 2.3, 3.2, 4.3 and 4; *p* = 0.0018, 0.0009, < 0.0001 and < 0.0001, respectively)
Ratcliffe et al. [34] 2016 Tanzania	Open Birth Days for pregnant women (complementing the antenatal care sessions) and RMC Workshops for providers (adapting the WHO Health Workers for Change curriculum)	Pre- and post-tests with participants in Open Birth Days (*n* = 362) and with attendants to the RMC Workshop (*n* = 76), direct labour observations (*n* = 459), structured community follow-up interviews (*n* = 149) and structured interviews with providers (*n* = 55)	During community follow-up interviews, 75.8% of women reported being very satisfied with their delivery experience compared to only 12.9% at baseline. At baseline, quality of care was rated as “excellent” (0%) or “very good” (2.9%), with an increase to 22.8% and 40.3% respectively at evaluation. Patient satisfaction with provision of health care improved, from 10% of women reporting “very satisfied” to 76.5%
Smith et al. [35] 2022 Zambia	BETTER pain management toolkit (Breathe, Encourage, Turn, Think, and Rub), including pain management technique posters, massage balls and a pain management manual with a partograph guide. Feedback box to empower clients to regularly assess clinic performance. Provider–client promise document on agreed behaviours during labour and delivery. Reflection workshop for providers to build an intention to change care as a facility introducing solutions. Fresh start funds to generate a sense of agency in changing the experience of care	Surveys with health facility providers (*n* = 33 and 35) and post-partum women (*n* = 60 and 92) before and after the intervention, interviews at endline (*n* = 5) and direct labour observations (*n* = 10). Each intervention site was matched to a similar comparison facility	Clients at implementation facilities were 15% less likely to experience any form of D&A compared to clients at comparison facilities (*p* = 0.01). Providers at intervention facilities reported greater use of more evidence-based pain management techniques at endline relative to baseline (*p* = 0.003). Though not statistically significant, findings suggested that providers in intervention facilities were more likely to be more empathetic towards clients (*p* = 0.07). Both clients and providers at intervention facilities found utility in the feedback box

*RMC* respectful maternity care, *D&A* disrespect and abuse, *OR* odds ratio, *Roots* black-owned culturally centred birth clinic, *BIPOC* black indigenous and people of colour, *GVtM* giving voice to mothers study

All the studies concluded that the implemented intervention resulted in an improvement in the care received by the delivering women. Kujawski et al. and Smith et al. reported 66% and 15% reduced odds of suffering D&A, respectively [31, 35]. Abuya et al. reported a decrease in D&A from 20 to 13% [28], and Asefa et al. found an 18% reduction in the number of experienced mistreatment components [30]. Afulani et al. observed a RMC increase from 12 to 64%, although their results differed from the other studies in that verbal and physical abuse paradoxically increased (despite the improvement in reports of being treated with respect) [29].

Oosthuizen et al. documented that different RMC components improved with the intervention [32], Molina et al. reported that satisfaction and the perceived quality of care improved [26], and for Gélinas et al. it was the way in which women were received at the health facility and the attitude of health professionals that were decisive for this level of satisfaction with care [33].

Ratcliffe et al. found that there was an increase in patient and provider knowledge of user rights, as well as women’s knowledge of the labour and delivery process and provider’s empathy for the women they served, with improved communication and user reports of satisfaction and perceptions of care quality [34]. Almanza et al. described that autonomy and respect scores were statistically higher for clients receiving culturally centered care at Roots, but no statistical significance was found in scores between black, indigenous and people of colour, and white clients [27]. More detailed results are presented at Table 2.

## Discussion

This scoping review synthetised 10 articles testing any kind of initiative specifically designed to reduce D&A or to promote RMC for women seeking care during childbirth in health facilities around the world.

Our results indicate that there are promising interventions to tackle this phenomenon. Even though it was a small sample of articles and in some cases the improvements were not extraordinary, they were sufficiently encouraging to implement context-specific programmes, to make the step from explanatory research to intervention and implementability.

Only 10 articles met the eligibility criteria. This points to a lack of evidence regarding initiatives specifically designed to tackle this phenomenon. Most of the efforts so far have been directed at determining the frequency of D&A and debating its terminology. This is especially relevant in high-income countries, as illustrated by the fact that all the interventions were studied in Africa, with the exceptions of Mexico [26] and the United States [27].

As noted before, childbearing women from middle and high-resource countries have also reported mistreatment and D&A during hospital births [8–14]. Although the evidence presented by this article can be of value for these higher-income settings, it is important to acknowledge that in many African countries or other developing nations, women’s social status is very low, they have less access to information and education, and live in very closed patriarchal societies, making them a vulnerable population. Therefore, investment on this type of approach could have a different impact in women’s lives in this context. Nevertheless, this should not restrain high and middle-income countries from implementing similar initiatives to the described in this study, since women in these higher-resource settings could also benefit from reducing mistreatment, D&A during childbirth and promoting RMC.

Most of the articles reviewed included training as a relevant part of the intervention. Every study that did so, concluded that it resulted in an improvement of the care received by the delivering women [26, 28–30, 32–35]. Physical abuse was the most consistently reduced [28, 30, 31]. These results suggest that provider education should include a form of RMC training, which should be encouraged by Gynecology and Obstetrics services.

In the case of Afulani et al. their results differed from the other studies in that verbal and physical abuse paradoxically increased (despite the improvement in reports of being treated with respect). A potential reason they found was that, while treating women with dignity and respect was emphasised in the training, verbal and physical abuse never actually occurred in their simulations, not giving a chance for improvement [29]. Relative to this, specific types of provider training should be assessed by further scientific research.

Effort should also be headed towards finding any other kind of tools that could complement or enhance these trainings when implemented. Other strategies that only a few articles explored included open maternity days [28, 34], clinical checklists [26], wall posters [30], and constant user feedback [28, 31, 32, 35]. While only tested by 1–4 studies each, every one of them seemed to complement the training effectively.

Most of the interventions addressed this issue from a RMC approach [26, 29, 30, 32, 35], especially apart from the ones centred on reducing mistreatment and D&A directly. This suggests that RMC constitutes the main initiative currently addressing women experiences of care during childbirth.

In the case of Asefa et al. although physical abuse was indeed reduced, no change was observed in the level of verbal abuse and neglect and discrimination, pointing to the fact that ingrained negative and normalised behaviours require time to change and are strongly associated with age and experience of service providers [30].

Evidence shows that women’s healthcare is profoundly influenced by sociocultural factors and entrenched gender norms. Health providers often incorporate their own beliefs and biases into their practices, which shape the care they deliver. Addressing these problems requires not only changing the attitudes of health professionals, but also confronting the broader sociocultural beliefs prevalent within communities. Without challenging and transforming these ingrained norms, efforts to improve women’s healthcare will continue to face significant obstacles [36, 37].

Relative to this, all the interventions were carried out at facility level, without directly addressing the structural determinants of health related to gender-discrimination at policy level, which although difficult to achieve, could potentially be more effective [16, 17]. Besides, efforts directed towards designing community level interventions should also be made.

Our results are similar to those described by Downe et al. In their systematic review [38], they analysed the articles by Abuya et al. [28], Kujawski et al. [31] and Ratcliffe et al. [34], and two other studies (one placed in South Africa only assessing birth companions, and another one in Sudan testing a communication-building package with staff). They found that RMC interventions increased women’s experiences of respectful care by almost four times, and reduced D&A by about two-thirds. In terms of specific attitudes and behaviours, they found that RMC initiatives could reduce physical abuse, with less evidence on other components of D&A. These results coincide with the ones presented in our study.

The articles included in our review shared several limitations. Most of them lacked a control group, which removed the ability to properly distinguish the intervention’s effect from other contextual factors during the implementation period. In addition, the majority of the initiatives were short (one took place during a year and a half [28], but the rest only lasted for a few months). Added to the fact noted before, that ingrained negative and normalised behaviours require time to change, this could have underestimated the potential effects of the interventions, but it also made it impossible to assess their long-term sustainability. Finally, for the articles that interviewed women as a means of intervention evaluation, social desirability and recall bias could have altered the results, and studies that included direct labour observations could have also been influenced by the Hawthorne effect (as observed providers may have acted more self-consciously).

Our study also has its own limitations. Being a scoping review, it lacked the degree of control that a systematic review could have offered. However, we felt that this allowed us to explore further findings, serving as a useful landscape analysis. PubMed was the only search engine screened, and we only considered articles with an abstract. Furthermore, given the changing terminology regarding this topic, a standardised search formula could not be used, which might have left some studies out of our scope. Nevertheless, we consider that most of the available evidence was reviewed within this article, providing a comprehensive approach regarding interventions to address this issue.

## Conclusion

The 10 articles reviewed in this study indicate that there are promising interventions to reduce D&A and promote RMC for women during facility-based childbirth. Provider training is the most proven strategy, and physical abuse the most consistently reduced. The specific types of training and different initiatives that complement them should be evaluated through further scientific research, and RMC interventions that apply these strategies should be implemented by health institutions. Beyond the need for further research and implementation of the actions already examined, there is an urgent need to establish and evaluate more structural interventions and policies, in order to modify the social and health contexts that impede full RMC to ensure a human rights-based maternity care for women giving birth in health facilities around the world.

## Data Availability

All data analysed during this study are included in the published articles cited.

## Abbreviations

D&A: Disrespect and Abuse
RMC: Respectful Maternity Care
WHO: World Health Organisation
