# Correction: Respectful maternity care interventions to address women mistreatment in childbirth: what has been done?

**DOI:** 10.1186/s12884-024-07003-y

**Published:** 2024-11-27

**Authors:** Pablo Mira‑Catala, Ildefonso Hernandez‑Aguado, Elisa Chilet‑Rosell

**Affiliations:** 1 Public Health Department, Miguel Hernandez University, Alicante, 03550 Spain; 2 CIBER de Epidemiologia y Salud Publica (CIBERESP), Madrid, 28029 Spain

**Correction: BMC Pregnancy Childbirth 24**,** 322 (2024)**

10.1186/s12884-024-06524-w

Following publication of the original article [1], the authors identified an error in the author names. The given names and family names were erroneously transposed.

The incorrect author names are: Mira‑Catala Pablo, Hernandez‑Aguado Ildefonso, Chilet‑Rosell Elisa.

The correct author name are: Pablo Mira‑Catala, Ildefonso Hernandez‑Aguado, Elisa Chilet‑Rosell.

The author group has been updated above and the original article [1] has been corrected.
